# Individual and country-level determinants of nursing home admission in the last year of life in Europe

**DOI:** 10.1371/journal.pone.0213787

**Published:** 2019-03-14

**Authors:** Erwin Stolz, Hannes Mayerl, Éva Rásky, Wolfgang Freidl

**Affiliations:** Institute of Social Medicine and Epidemiology, Medical University of Graz, Graz, Austria; BITS Pilani, INDIA

## Abstract

**Background:**

Previous research has focussed on individual-level determinants of nursing home admission (NHA), although substantial variation in the prevalence of NHA between European countries suggests a substantial impact of country of residence. The aim of this analysis was to assess individual-level determinants and the role of country of residence and specifically a country`s public institutional long-term care infrastructure on proxy-reported NHA in the last year of life.

**Methods:**

We analysed data from 7,018 deceased respondents (65+) of the Survey of Health, Ageing and Retirement in Europe (2004–2015, 16 countries) using Bayesian hierarchical logistic regression analysis in order to model proxy-reported NHA.

**Results:**

In total, 14% of the general older population utilised nursing home care in the last year of life but there was substantial variation across countries (range = 2–30%). On the individual-level, need factors such as functional and cognitive impairment were the strongest predictors of NHA. In total, 18% of the variance of NHA was located at the country-level; public expenditure on institutional care strongly affected the chance of NHA in the last year of life.

**Conclusion:**

On the individual-level, the strong impact of need factors indicated equitable access to NHA, whereas differences in public spending for institutional care indicated inequitable access across European countries.

## Introduction

Against the backdrop of ageing societies in Europe, research on determinants of nursing home admission (NHA) in old age is important in order to assess what drives costly institutionalisation at the end of life and to which degree the comprehensive personal care services provided therein are allocated according to need (= equity)[1]. Two meta-analyses[2,3] found evidence for the following risk factors of NHA (classified according to[1]): older age (predisposing); living alone, low social support, being a tenant rather than a house owner (enabling); poor self-rated health, functional impairment, cognitive impairment, dementia, a high number of prescriptions, prior NHA (need factors). In contrast to these well-documented individual-level risk factors, we are not aware of any cross-national studies assessing the role of country-level characteristics for NHA, as existing studies have focussed exclusively on individual-level determinants (e.g.[4–10]). This is all the more surprising, since national differences in public expenditure and private out-of-pocket costs with regard to long-term institutional care in Europe have been repeatedly reported to be substantial[11–14]. For example, public expenditure on institutional care (2010) in Greece or Estonia was estimated to be below 0.2% of their gross domestic product (GDP), whereas this amounted to around 2% in Sweden or the Netherlands[15]. These differences in public expenditure result in a highly varied nursing and care home infrastructure[16], which ought to affect the chance of NHA in the last year of life. A recent study focussing on inpatient care facilities which includes hospital and hospice next to nursing home admissions, for example, reported considerable between-country variation in such admissions and suggested that these are at least partly driven by system-level or cultural factors[17]. Finally, it is unknown, whether or not the impact of the well-established individual-level determinants varies between countries, and if, whether this variation is linked to a country’s public institutional care infrastructure. For example, older adults in more generous public long-term care systems might access nursing home care sooner, that is, with fewer functional limitations compared to older adults in countries with a more restricted public institutional long-term care infrastructure.

The lack of cross-national research on NHA is likely due to both restricted data availability and methodological issues. First, cross-national comparable individual-level datasets on institutionalised older adults[18] or NHA[19] are exceedingly rare and not representative for the general older population. Second, the estimation of variance components and country-level effects is difficult when data is available only for a handful of countries[20–22].

Against this backdrop, we analyse cross-national data on the living situation in the last year of life when care need is highest and use of nursing home care services most prevalent[23–26]. Based on data from both the deceased respondent and proxy-interviews, we used Bayesian hierarchical logistic regression analysis to model (1) the impact of individual-level determinants on proxy-reported NHA in the last year of life, and (2) their variation between countries, as well as (3) the impact of country-level public spending on proxy-reported NHA and (4) cross-level interaction effects between country-level spending and individual-level predictors.

## Methods

### Data

The Survey of Health, Ageing and Retirement in Europe (SHARE) provides representative data of older community-dwelling adults in Europe. Data on deceased respondents (aged 65 or over) who participated in one or more of six panel waves of SHARE (2004/2005, 2006/2007, 2008/2009, 2010/2011, 2013, 2015) are available for 16 European countries: Austria, Belgium, Czech Republic, Denmark, Estonia, France, Germany, Greece, Italy, Netherlands, Poland, Portugal, Slovenia, Sweden and Switzerland. End-of-life interviews with proxy respondents were conducted in 7,018 (83.5%) cases out of 8,406 deceased respondents (2004–2013).

### Variables

Information on NHA stemmed from proxy-respondents while information for predictor variables was either self-reported, that is, stemming from the deceased respondent him-/herself in the previous wave or proxy-reported after the death of the SHARE respondent.

Nursing home admission (NHA): Proxy respondents were asked: ‘Has [{Name of deceased}] had any care in a nursing home in the last 12 month of [his/her] life?’. If proxy-respondents answered ‘yes’, NHA was coded 1, otherwise it was coded 0.

Predisposing Factors: Sex, age of death (in years), and education (ISCED97: low = 0-2/high = 3–6).

### Enabling factors

This set of predictors included social and material resources at the individual- and country-level. The information on the area of living (rural, village, small town/large town, suburbs, city), living alone (no/yes), and income-poverty (<60% of national median net household income) (no/yes) stemmed from the deceased respondent. From the proxy interviews, receipt of informal care (no/yes), use of professional home care services (no/yes), and homeownership (no/yes) were considered. On the country-level, we included the public expenditure for institutional long-term care services in percent of the GDP ([15] p.10; for Switzerland [11] p.48, 232) as a proxy measure for the level of public provision of institutional long-term care services.

### Need factors

Based on interviews with the deceased respondent, poor self-rated health (no/yes) and cognitive functioning, assessed by an adapted ten-word delay recall test with scores ranging from 0–10[27], were included. From proxy-respondents, information on the number of functional limitations in the last 12 months (basic and instrumental activities of daily living: dressing, walking across a room, bathing/showering, eating, getting in/out of bed, using the toilet, preparing a hot meal, shopping for groceries, making telephone calls, taking medication; 0–10), on the duration of received care/help in the last 12 months (<6 months/≥ 6 months) and on requiring 24-hour care/help (no/yes) was incorporated. Additionally, proxy-respondents provided information on cognitive problems of the deceased respondent, that is, whether he/she had (no/yes) any difficulty recognizing family members or good friends in the last year of life lasting longer than 3 months.

### Model

All data analyses were performed using *R*: *A language and environment for statistical computing* (v3.3.2). Hierarchical logistic regression analysis was used to model the impact of predictors on proxy-reported NHA among older adults (level 1) nested in countries (level 2). Due to the small number of countries (n = 16), a Bayesian estimation procedure (Hamiltonian Monte Carlo; weakly informative priors, three chains, each 5,000 iterations) was applied instead of standard maximum likelihood[20–22] using R-package *brms* (v1.3.1)[28], a frontend for S*tan* (v2.14.1)[29]. Odds ratios (OR) based on the mean posterior distribution and 95-% credible intervals (CI) indicate effect size and estimated uncertainty. The following model types were fitted: (1) Random intercept-only (M0) to evaluate total country-level variation in NHA; (2) random intercept models of predisposing/need factors (M1a) and enabling factors (M1b) separately in order to assess the relative importance, and all individual-level predictors combined (M1c); (3) intercept-as-outcome model (M2) in order to assess the role of public expenditure on individual-level NHA; (4) random-intercept-random-slope models in order to assess potential cross-national variation of individual-level effects separately (M3a-M3p) and combined (M4). Finally, cross-level interaction terms with public expenditure were included for those predictors which’s effect varied across countries (M5). Model fits were assessed with the Watanabe-Akaike-Information Criterion (WAIC)[30] and marginal R^2^[31] (maximum-likelihood estimation).

### Missing values

Characteristics of deceased respondents for which proxy-interviews could not be realised (N = 1,388, 16.5%) were compared with those for whom proxy-interviews were successfully conducted. It showed that missing end-of-life interviews were more likely realised for deceased older men (18.7%) than women (14.0%; χ^2^ = 33.5, df = 1, p<0.001), more likely available for those who lived with others (19.5%) rather than alone (9.7%; χ^2^ = 123, df = 1, p<0.001) and for those with high (22.9%) compared to those with a low level of education (12.8%, χ^2^ = 139, df = 1, p<0.001).

Among realised end-of-life interviews (N = 7,018), missing values due to item non-response were <5% per variable, except for problems recognizing family members or good friends (7.8%) which was not included in the first round of end-of-life interviews (2006/07), and the outcome (NHA: 6.3%), which was due to an error in the filter question in the sixth wave of SHARE (2015). Missing values were considered missing-at-random (MAR)[32] and a Random Forest imputation procedure (R-package *missForest*, v2.23)[33–34] was used to impute missing data.

### Ethical approval

The study was carried out in compliance with the principles laid down in the Helsinki Declaration. No children were included in the study sample. Informed consent was obtained from all respondents in SHARE. The SHARE study is subject to continuous ethics review. During Waves 1 to 4, SHARE was reviewed and approved by the Ethics Committee of the University of Mannheim. Wave 4 and the continuation of the project were reviewed and approved by the Ethics Council of the Max Planck Society. In addition, the country implementations of SHARE were reviewed and approved by the respective ethics committees or institutional review boards whenever this was required.

## Results

51.8% of the total sample were men, and the average age at death was 79.9 (SD = 7.8, range = 65–104) for men and 82.7 (SD = 8.4, range = 65–104) for women. Sample size by country varied between 94 in Slovenia (waves 4–6 only) and 961 in Spain. Proxy-respondents were mostly partner (39.8%) or children (32.8%) of the deceased.

**Fig 1:**
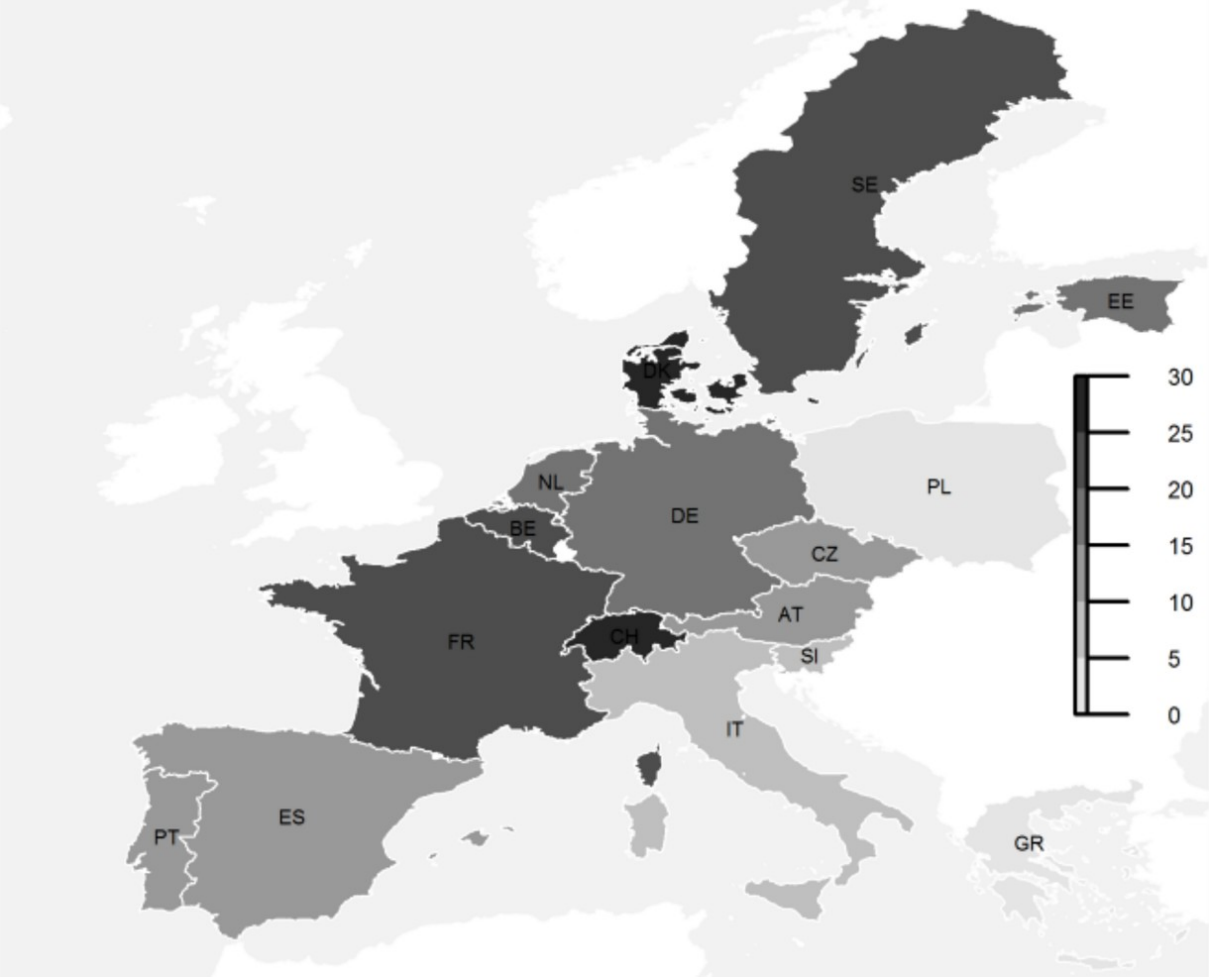
Prevalence of nursing home admission in the last year of life (in %). SHARE, waves 1–6 (v5.00, v6.00), unweighted data, n = 7,018.

In total, NHA in the last 12 months of life was reported for 14.3% (n = 1,049) of the sampled older adults (65+). Country differences in NHA were substantial (Chi^2^ = 341, df = 15, p<0.001), ranging from 2.1% in Greece and 3.5% in Poland to 27.9% in Switzerland and 29.7% in Denmark. Geographically, a North-West/South-East gradient in NHA in the last year of life was apparent (Fig 1), with Switzerland as an exception.

17.9% of the total variance of NHA was located on the country level (M0). Including all individual-level variables (M1c) did not reduce the intra-class-correlation coefficient, which indicated that the substantial country-level differences in the prevalence of NHA were not due to compositional effects. R^2^ was 0.17 in the model including only individual-level predisposing and need factors (M1a), 0.05 for individual-level enabling factors only (M1b), and 0.18 when all individual-level predictors were included (M1c). Including public expenditure for institutional care as a country-level predictor (M2) decreased country-level variance (14.0%) and increased R^2^ (= 0.24) considerably, implying a contextual effect.

The third column in Table 1 shows the results of the logistic regression analysis of individual-level characteristics and country-level public expenditure on the chance of NHA in the last year of life. Respondents who died in older age were more likely of being admitted to a nursing home in the last year of life compared to those who died earlier. With regard to enabling factors, living alone increased the chance of NHA by 76%, whereas receiving informal care, being a home owner and falling below the poverty-risk threshold decreased the chance of NHA by 39%, 22% and 20% respectively. The strongest effects of individual-level predictors showed for the need factors. Older adults with a high level of functional impairment (+141%), who were not able to recognize family members or friends for a prolonged period of time (+86%), who required prolonged (+74%) or intensive 24-hour (+50%) long-term care in the last year of life had a considerably higher chance of NHA than their respective counterparts. Finally, in countries which provide extensive public institutional care services such as Sweden or the Netherlands, chances for NHA in the last year of life are almost 2.5 times higher than in countries with restricted public spending on institutional care such as Portugal, Greece or Poland.

**Table 1:** Individual-level sample characteristics and results from hierarchical logistic regression analysis of nursing home admission (NHA).

	Sample characteristics	Logistic regression (NHA = 1)
	n (%) / n (mean, SD)	OR (95%-CI)
PREDISPOSING		
Sex: Male ^SR^	3,637 (51.8)	1
Sex: Female ^SR^	3,381 (48.2)	1.13 (0.96–1.32)
Age at death (years) ^PR^	7,018 (81.2, 8.2)	1.49 (1.26–1.76)
Education: low ^SR^	4,617 (67.5)	1
Education: high ^SR^	2,223 (32.5)	1.08 (0.91–1.29)
ENABLING		
Location: rural/village/small town ^SR^	3,637 (53.8)	1
Location: large town/suburb/city ^SR^	3,118 (46.2)	1.02 (0.88–1.19)
Living alone: no ^SR^	4,646 (66.7)	1
Living alone: yes ^SR^	2,318 (33.3)	1.76 (1.49–2.07)
Home care services: no ^PR^	4,252 (61.3)	1
Home care services: yes ^PR^	2,680 (38.7)	0.95 (0.82–1.12)
Informal care: no ^PR^	3,662 (52.2)	1
Informal care: yes ^PR^	3,356 (47.8)	0.72 (0.60–0.85)
Income poverty: no ^SR^	5,028 (71.6)	1
Income poverty: yes ^SR^	1,936 (27.6)	0.83 (0.70–0.98)
Home owner: no ^PR^	2,838 (41.2)	1
Home owner: yes ^PR^	4,049 (58.8)	0.82 (0.70–0.96)
Country-level public expenditure	7,018/16 (0.76, 0.59)	2.39 (1.21–4.70)
NEED		
Poor SRH: no ^SR^	4,456 (64.8)	1
Poor SRH: yes ^SR^	2,419 (35.2)	1.16 (0.99–1.36)
Cognitive functioning ^SR^	6,964 (2.1, 1.9)	0.74 (0.63–0.87)
Problems recognizing family/friends: no ^PR^	5,300 (82.0)	1
Problems recognizing family/friends: yes ^PR^	1,167 (18.1)	1.86 (1.57–2.22)
Functional impairment ^PR^	6,820 (5.0, 4.7)	2.41 (1.96–2.97)
24-hour care need: no ^PR^	5,494 (81.8)	1
24-hour care need: yes ^PR^	1,225 (18.2)	1.48 (1.23–1.78)
Duration of care provision: < 6 months ^PR^	3,835 (54.7)	1
Duration of care provision: ≥ 6 months ^PR^	3,173 (45.3)	1.63 (1.34–1.97)

Survey of Health, Ageing and Retirement in Europe (SHARE), waves 1–6 (v5.00, v6.00), n = 7,018, unweighted data. NHA = nursing home admission, SD = standard deviation, OR = odds ratio, 95%-CI = 95% credible interval, SRH = self-rated health, SR = self-reported, PR = proxy-reported. Reported odds ratios refer to the intercept-as-outcome model (M2). All numeric predictor variables were mean-centered and divided by 2 standard deviations in order to make them more easily comparable with effect sizes from binary categorical variables.

Cross-national variation of the impact of individual-level predictors was clearly supported by substantially improved model fit (ΔWAIC) in relation to its standard error upon including single random slopes for four predictor variables: problems recognising family members or friends (ΔWAIC = -35.7, SE_ΔWAIC_ = 12.8), home care services (ΔWAIC = -29.2, SE_ΔWAIC_ = 10.9), informal care (ΔWAIC = -22.3, SE_ΔWAIC_ = 10.3) and 24-hour care need (ΔWAIC = -20.1, SE_ΔWAIC_ = 9.6). It showed, for example, that the effect of cognitive problems on NHA was strongest in Germany and weakest in Italy. The effect of home care services on NHA services was clearly negative in Greece or Austria but positive in Estonia, Portugal or Denmark. Informal care, finally, had a negative effect on NHA in most countries, particularly so in Greece and Spain, whereas the effect was positive in Sweden and Switzerland. Cross-level interaction terms with country-level public expenditure showed 95%-credible intervals not overlapping with zero (respectively with one in case of ORs) only with regard to informal care (OR = 1.86, CI = 1.21–2.86). This statistically significant and positive cross-level interaction means that in countries with low public spending on institutional care, informal care is a stronger impediment for NHA than in countries with more established public financing of institutional care.

## Discussion

In this study, we analysed cross-national data from deceased survey respondents and from post-mortem proxy-interviews from 16 European countries using Bayesian hierarchical logistic regression models. We assessed both the impact of individual-level predisposing, enabling and need factors as well as of country of residence, respectively public long-term care expenditure on institutional care on proxy-reported NHA in the last year of life. We are not aware of any other study attempting to assess the impact of country-level characteristics on NHA using nationally-representative data from multiple countries so far. Assessing determinants of NHA in the last year of life is important, as institutional nursing care is the most expensive type of long-term care for the public, the clients and their relatives[11,12,14,15]. Access to nursing homes should be equitable[1], that is, primarily according to the need for care.

The country of residence played a major role with regard to NHA in the last year of life, as approximately one fifth of the variation of NHA was located on the country-level. In short, *where* people age and spend their last year of life is itself a powerful determinant of NHA in Europe. This adds to the existing literature on predictors of institutionalisation ignoring this relevant contextual enabling factor[1] so far (e.g.[3]). A significant share of the impact of the parameter country of residence was explained by substantial differences in public spending on institutional long-term care[11,12,15,35]. Thus, individuals with highly similar levels of care need may have a profoundly different chance to use nursing home services in their last year of life because they happen to live in countries with starkly varying levels of public protection and service provision. This scrutinises the notion of equitable access to professional long-term care services across European countries in the last year of life, a time period in an adult’s life when the need for care from others to complete even the most basic activities of daily living is highest[23–26].

With regard to the effects of individual-level determinants of NHA, our results correspond to those of previous single studies (e.g.[4–10]) and two meta-analyses[2,3], highlighting the role need factors such as cognitive and functional impairment. The impact of most individual-level predictors tested did not vary substantially across countries (see also[36]); exceptions included the provision of professional home care services as well as informal care provided by family members and others.

Comparison of the impact of individual-level need versus enabling factors showed that NHA was mostly driven by need factors[3], which supports the notion of a largely equitable allocation of nursing home services to those with the highest care need[1], at least at the individual-level, that is, within a given country. Enabling factors, that is, not living alone, receiving informal care, being a home owner or being at poverty-risk decreased the chance of NHA. This was independent of need factors, which is in line with results from two recent studies from Scandinavia[9,10]. In our study, utilisation of home care services was not uniformly associated with NHA in the last year of life, as for example a clearly negative effect showed for Greece compared to a positive one in Denmark. This could reflect country differences in the interlocking (or a lack thereof) of professional home care and institutional care services, that is, whether home care services precede or substitute for nursing home care. The two-faced role of home care services as a determinant of NHA is reflected in the literature[3]–i.e. as a positive or negative risk factor for NHA–and could also be due to the underlying modalities, that is, whether it is mostly publicly provided and thus free of substantial charges[7] or mostly privately paid[8]. Thus, future research should aim to combine more detailed empirical analysis of long-term care policies and regulations on the country-level with cross-national individual-level survey data in order to assess the role of different care-regimes[37].

The negative effect of the availability of informal caregivers on NHA found in this study is also in line with previous findings[2–4]. Interestingly, there was a notable variation of this effect across countries–stronger and negative in the South- and Eastern European countries such as Spain, Greece or Slovenia, and even positive in Switzerland and Sweden–which could be related to the inconsistent results reported in a previous meta-analysis[3]. According to our study, this cross-national variation was in part due to substantial differences in the level of public institutional care provision[11]. This can be interpreted as such that institutional care provision in nursing homes in Southern and Eastern European countries–which is marginal in these countries and represents a form of long-term care preferred by very few[38]–is reserved as a residual, last option for those (unfortunate) older adults without family members willing or capable of providing the intensive care often required at the end-of-life. In North-western Europe in contrast, nursing homes represent a more viable option in case of substantial care need. There, informal care provision and nursing home care tend to be less mutually exclusive and more likely successive types of care provision[39], depending on care need.

In conclusion, this study demonstrated that next to established individual-level need factors, country of residence respectively public spending on institutional care represents a decisive, yet hitherto neglected predictor of NHA at the end of life among older Europeans. Thus, caution should be taken when results on determinants of NHA from single-country studies are summarised or subject to a meta-analysis, particularly with regard to varying effects of informal care and professional home care. Our results also imply that discussions on the equitability of comprehensive and expensive nursing home care at the end of life should not end at national borders but take the substantial variation between European countries into account. The strengths of this study are the utilisation of cross-national representative and comparable survey data, usage of appropriate statistical procedures and a large number of individual-level predictors. Furthermore, the use of a combination of self- *and* proxy-reported information mitigated the problem of censoring often associated with surveying older adults in their last years of life[40]. At the same time, proxy-interviews may also constitute a limitation since different proxy-interviewees may provide differently accurate accounts of the living situation in the last year of life of the deceased. A further limitation is the potential selectivity of end-of-life interviews with proxy-respondents. However, the number of missing end-of-life interviews was reasonably low and patterns of unit non-response suggest that, although the prevalence rate of NHA reported in this study could be somewhat upward-biased, the impact on the results with regard to the determinants of NHA is probably limited. A final limitation of this study is that the length of the stay in nursing homes was unknown, although the duration has been suggested to be relevant in itself and with regard to the associated determinants[41].
